# Academic outcomes of inclusive education in typically developing children

**DOI:** 10.3389/fpsyg.2024.1448935

**Published:** 2025-01-07

**Authors:** Andrijana Bakoč, Branislav Brojčin, Slobodan Banković, Nenad Glumbić, Mirjana Đorđević

**Affiliations:** ^1^Faculty of Medicine Foča, University of East Sarajevo, East Sarajevo, Bosnia and Herzegovina; ^2^Faculty of Special Education and Rehabilitation, University of Belgrade, Belgrade, Serbia

**Keywords:** knowledge test, intellectual disabilities, mathematics, general knowledge, school inclusiveness, teacher characteristics

## Abstract

**Introduction:**

The fact that inclusive education has existed in Bosnia and Herzegovina for twenty years opens the question of how it affects typically developing children, among other things. This paper aims to examine the differences in general knowledge and mathematics of typically developing students with regard to whether they attend classes that include students with intellectual disabilities or not, as well as to determine the relationship of their knowledge with teachers’ characteristics and the inclusiveness of schools they attend.

**Methods:**

The sample included 331 students from 18 regular elementary school classes. The sample was divided into two subsamples – respondents who attended classes that included students with intellectual disabilities and respondents who attended classes that did not include any students with disabilities. The *Peabody Individual Achievement Test – revised* was used to assess general knowledge and mathematics, while *My Thinking About Inclusion Scale*, *Bender Classroom Structure Questionnaire, Teacher Efficacy for Inclusive Practices,* and *Inclusive Process Evaluation Scale* were used for assessing the teacher and school variables.

**Results:**

No statistically significant difference was found between the two subsamples. The schools’ inclusiveness was related to better achievements of all respondents, mainly in terms of work organization. The examined teachers’ characteristics established different relationships with academic achievements in different subsamples.

**Conclusion:**

Attending classes with a student with intellectual disability did not negatively affect the academic achievements of other students in class. The school’s inclusiveness in terms of work organization was positively related to the academic achievement of all students, while the relation of teachers’ characteristics with students’ achievements is a complex phenomenon that requires further research. Given the results obtained, to achieve better academic outcomes, teachers in classrooms attended by students with intellectual disability should be encouraged to use metacognitive and individualized teaching strategies. Furthermore, school principals and school administration would contribute to the success of all students by organizing the school on the principles of inclusion.

## 1 Introduction

Inclusive education aims to increase the inclusion of all children into the regular education system. Opertti et al. (2014) believe that four fundamental ideas have influenced the development of inclusive education internationally: first, the educational practice based on human rights; then, improving the learning conditions for children with disabilities and later for all marginalized groups; and finally, developing the educational system that will be able to provide quality education for all. With regard to this, there has been a paradigm change that involves shifting the focus on organizations and asking questions about how they themselves make barriers to inclusion and how schools can provide equal education for all children (Paseka and Schwab, 2020).

In relation to students with disabilities, inclusion advocates believe that academic achievements in an inclusive environment will be better due to higher expectations in regular classes (Cole et al., 2004; Van Mieghem et al., 2020) and that adapted instruction and support in inclusive classes may positively affect other students (Ruijs and Peetsma, 2009). Two recent meta-analyzes have found that teachers in different countries have positive attitudes toward inclusive education. Examining the attitudes of teachers from 36 different countries toward this educational approach, Van Steen and Wilson (2020) determined that the attitudes of teachers in two-thirds of analyzed studies (44 out of 64 samples) were positive. Similarly, Guillemot et al. (2022), compiling the results of 131 studies from 55 different countries published between 2000 and 2021, report that teachers globally hold positive attitudes toward inclusive education. For example, Pakistani teachers believe that inclusive education is a desirable practice and that all students have a right to be educated in regular classes (Khan et al., 2017), while German teachers believe that the learning characteristics of all children should be taken into account in the teaching process (Hellmich et al., 2019). Turkish teachers agree that both typically developing students and their peers with disabilities benefit from this education approach (Sakiz and Woods, 2014), while Greek teachers recognize that interaction among different groups of students fosters understanding and accepting diversity (Galaterou and Antoniou, 2017). Among other countries, predominantly positive teachers’ attitudes are found in China (Fu et al., 2023), Bosnia and Herzegovina (Memisevic et al., 2024), Spain (Lacruz-Pérez et al., 2021), and Latvia (Pavitola et al., 2019). However, some teachers are concerned that academic standards and students’ achievements may be lowered (Sharma and Desai, 2002; Sokal and Sharma, 2014; Yadav et al., 2015). In addition, some teachers believe they will have more obligations (Saloviita, 2020b) and that children with disabilities will require great effort and time, which may lower the overall outcomes (Dyson et al., 2004).

On the other hand, parents of typically developing children understand that their children may benefit from using different education approaches in an inclusive environment (Vlachou et al., 2016). They also see the advantages of inclusive education in respecting and accepting diversity. However, some of them are concerned that their children may lag behind in their educational progress due to the attention children with disabilities require (Tafa and Manolitsis, 2003).

Intellectual disability (intellectual developmental disorders; disorders of intellectual development) is a condition that occurs in the developmental period and is characterized by significant limitations in both intellectual functioning and adaptive behavior (American Psychiatric Association, 2022; World Health Organization, 2024). Although students with moderate, severe, and complex disabilities have better academic achievements in inclusive compared to segregated settings, or their achievements are at least equal in both settings (Dell’Anna et al., 2022; Dessemontet et al., 2012), a paradox is observed that the inclusion of students with intellectual disability is a much slower process compared to the inclusion of students with other disabilities. Practitioners believe that it is hard, or even impossible, to include students with intellectual disability in regular education (Avramidis and Norwich, 2002; Lindner et al., 2023; Scruggs and Mastropieri, 1996). That is why it is particularly important to examine how classes that include students with intellectual disability function, including the area of academic functioning.

Education is a complex system whose success depends on the interaction of numerous subsystems. Thus, the outcomes of inclusive education, including academic ones, are, among others, influenced by school factors, such as the inclusiveness of the school, which provides a foundation for a safe and stimulating community and the development of shared values, but also ensures that inclusion permeates all school plans and encourages the participation of students and school staff, as well as developing an inclusive practice that reflects the school’s inclusive culture and policy (Booth and Ainscow, 2002). Naturally, the outcomes of inclusive education also depend on what happens in the classroom, where teachers’ characteristics play a significant role (e.g., attitudes toward inclusive education, self-efficacy for working in such an environment, and teaching methods they use).

Although inclusion is firmly based on ethical and moral principles, the challenge remains in finding concrete evidence to support its practical use. Kefallinou et al. (2020) state that the existing reviews of research evidence in support of inclusion are not sufficiently convincing. Evidence regarding the academic outcomes of inclusive education for typically developing students, the associated factors, and especially the influence of specific student groups is no exception.

This research was conducted in the Republic of Srpska, one of the two entities of Bosnia and Herzegovina, a Southeast European country. The education of children with disabilities is an integral part of the educational system and is implemented in regular schools, special classes in regular schools, and special schools for students with disabilities if that is assessed to be in their best interest. Additional services children receive in regular schools are not entirely satisfactory. Relevant research has not yet been conducted, and anecdotal data suggests that regular schools have insufficient resources.

Previous research does not provide a clear picture regarding the academic outcomes of typically developing students in inclusive education. On the other hand, students with intellectual disability are at particular risk of exclusion from regular classrooms (Buchner et al., 2021). With regard to this, the overall objective of this research was to examine the differences in general knowledge and mathematics of typically developing students depending on whether or not they attend classes that include students with intellectual disabilities. In addition, a specific objective of this study was to determine the relationship between the academic achievement of typically developing students in an inclusive environment and the inclusiveness of the schools they attend viewed through their inclusive organization and inclusive teaching. Finally, another specific objective of this research was to examine the relationship between general knowledge and mathematics achievements of typically developing students and teacher characteristics, such as attitudes toward inclusive education, self-efficacy for inclusive education, and the methods they use in teaching.

The main questions addressed in this study are: (1) Do typically developing students who attend classes with a student with intellectual disability differ in general knowledge and mathematics from those who attend classes that do not include students with disabilities? (2) Do students who attend more inclusive schools in terms of organization and teaching have higher achievements in general knowledge and mathematics? (3) Do students whose teachers have more positive attitudes toward inclusive education have better achievements in assessing general knowledge and mathematics? (4) Do students whose teachers have higher self-efficacy for inclusive education have better achievements in assessing general knowledge and mathematics? (5) Do students whose teachers more frequently use differentiated instruction and metacognitive strategies have higher achievements in general knowledge and mathematics?

From a theoretical perspective, the data obtained in this study will contribute to the body of knowledge that will address some authors’ beliefs that the academic achievements of all students (regardless of their characteristics) will be better in an inclusive environment than in traditional classrooms and schools (Demirdis, 2022; Freeman and Alkin, 2000; Ruijs and Peetsma, 2009; Vyas, 2022). Also, in accordance with Bronfenbrenner’s ecological model, we will examine the relationship between the classroom factors (the characteristics of instruction and teachers) and the school factors (inclusive organization and inclusive teaching) and student achievement in the assessment of general knowledge and mathematics.

From a practical perspective, the results of this research will help resolve parents and teachers’ concerns regarding whether these students will receive lower-quality instruction and lower educational standards than their peers who do not have classmates with disabilities (Ahsan et al., 2012; Dyson et al., 2004; Ruijs and Peetsma, 2009; Tafa and Manolitsis, 2003). Furthermore, the findings will help develop future teacher training programs. Data on the relationship between school inclusivity (in terms of teaching and organization) and academic achievement in the examined fields may serve as a guide and provide arguments for school administrations and decision-makers in directing the future development of schools toward greater inclusivity.

This study consists of five sections. The “Introduction” section is followed by “Literature review,” and “Theoretical background” sections. The “Method” section provides a detailed description of the sample, instruments, research procedure, and statistical method. The “Results” section presents and analyzes the collected data, while comparisons with other studies and possible interpretations of the findings are provided in the “Discussion” section. The practical significance of the results is given in the “Managerial implications” section, which is followed by “Limitations and future research avenues.” Finally, the “Conclusion” section highlights the key findings, recommendations, and the main contribution of this research compared to the literature.

## 2 Literature review

### 2.1 Students with intellectual disability in an inclusive environment – risk of exclusion

Students with intellectual disability, especially those with severe or profound intellectual disability, are at a particularly high risk of being excluded from regular and referred to segregated education (Cornelius and Balakrishnan, 2012; Ebersold et al., 2011; Ferguson, 2008). Buchner et al. (2021) explain this through a strong system of special schools and a firmly established belief that special schools provide better educational support to some students. In addition, they see responsibility in teacher education that is not aimed at meeting individual student needs, as well as in the competitive discourse of schools. These students are not sufficiently engaged or encouraged in an inclusive environment and lack academic self-confidence, which affects their in-class participation and inadequate academic experience. They do not have relationships with other students, are considered weird, and feel like outsiders (Sigstad, 2017).

### 2.2 Academic outcomes of inclusive education for typically developing students

The results of studies dealing with academic outcomes of inclusive education most often determine a neutral effect of inclusive education on the academic achievement of typically developing students (Dessemontet and Bless, 2013; Farrell et al., 2007; Kalambouka et al., 2007; McDonnell et al., 2003; Ruijs et al., 2010; Ruijs, 2017). Studies that obtain positive results are somewhat scarcer. They most often find significant but small effects (Demeris et al., 2007; Szumski et al., 2017), record more positive than neutral outcomes (Brady, 2010), and only occasionally indicate a clear, significantly greater academic progress (Cole et al., 2004). Still, studies that conclude that inclusive education negatively affects the academic achievement of typically developing students are the scarcest, and they, as a rule, refer to classes that include students with emotional and behavioral disorders (Fletcher, 2010; Kristoffersen et al., 2015). Also, some studies obtain results that are mixed, differentiated, or do not lead to clear conclusions (Daniel and King, 1997; Kart and Kart, 2021; Krammer et al., 2021; Spence, 2010).

Therefore, we hypothesize:

*Hypothesis 1:* Student achievement in general knowledge and mathematics will not be significantly related to the presence of students with intellectual disability in the classroom.

### 2.3 School inclusiveness

The Cambridge Dictionary (2013) defines *inclusiveness* as the quality of including many different types of people and treating them all fairly and equally. On the other hand, Ainscow and César (2006) state that the lack of organizational changes in schools is one of the biggest barriers to inclusive education policies (Ainscow and César, 2006). Similarly, Booth and Ainscow (2002) believe that changes in the ways of thinking and organization of specific schools may achieve a more inclusive education. With regard to that, school inclusiveness in this paper is viewed as the extent to which schools succeed in removing barriers in terms of organization and teaching to provide appropriate education for all their students.

Therefore, we hypothesize:

*Hypothesis 2:* Student achievement in general knowledge and mathematics will be positively related to school inclusiveness in terms of organization and teaching.

### 2.4 Teacher attitudes

Teachers’ attitudes toward inclusive education refer to their beliefs and feelings about including children with different educational needs in regular classes (Yada et al., 2022) and are believed to play a key role in implementing this educational approach (Gal et al., 2010; Saloviita, 2020a; Van Mieghem et al., 2020). These attitudes are closely related to teachers’ professional identities and competencies, thus affecting instruction and teacher behavior and contributing to learning (Heyder et al., 2020). Teachers’ attitudes toward inclusion are associated with positive or negative expectations and behaviors, which are in turn associated with teachers’ inclinations to use successful inclusive practices, which is ultimately reflected in student achievement (Hellmich et al., 2019; Pit-ten Cate et al., 2018; Sharma and Sokal, 2016; Schwab and Alnahdi, 2020). Furthermore, students whose teachers have more positive attitudes toward including students with special educational needs in the regular educational environment state that there is more satisfaction and cohesiveness in their classes, as well as fewer quarrels, competitiveness, and difficulties compared to classes whose teachers have less positive attitudes toward this issue (Monsen et al., 2014). Turkish math teachers believe that teachers’ positive attitudes toward the inclusion of students with special educational needs positively affect the academic achievement of all students, while negative attitudes affect not only the students with special educational needs but all students in an inclusive environment (Demirdis, 2022). Despite these beliefs and cited research results, quantitative empirical evidence directly examining the relationship between teacher attitudes toward inclusive education and student academic achievement is scarce (Heyder et al., 2020).

Therefore, we hypothesize:

*Hypothesis 3:* Student achievement in general knowledge and mathematics will be positively related to teachers’ attitudes toward inclusive education.

### 2.5 Teacher self-efficacy

Teacher self-efficacy can be viewed as teachers’ beliefs that they can affect their students’ performance, even in difficult or unmotivated students (Savolainen et al., 2012), and is considered crucial for the successful implementation of a good education system (Yada et al., 2022). In inclusive education, teachers with higher self-efficacy use inclusive teaching practices more often (Schwab and Alnahdi, 2020), are more patient with students with difficulties and criticize them less frequently (Gibson and Dembo, 1984), are more tolerant of student problematic behaviors and refer them less frequently to special education referral (Meijer and Foster, 1988). Teachers with high self-efficacy have more positive attitudes toward inclusion (Savolainen et al., 2012; Weisel and Dror, 2006), while low self-efficacy is associated with high levels of teacher anxiety toward including students with disabilities in their classes (Soodak et al., 1998).

Therefore, we hypothesize:

*Hypothesis 4:* Student achievement in general knowledge and mathematics will be positively related to teachers’ self-efficacy for inclusive education.

### 2.6 Differentiation and metacognitive strategies

Inclusive education requires not only access to the regular education system but educational justice for all, which requires teachers to create an educational environment that provides a stimulating teaching and learning process for all students (Lindner and Schwab, 2020). Differentiated instruction is based on the fact that variability exists in any group of students, and, therefore, teachers must expect diversity and adjust their teaching accordingly (Smit and Humpert, 2012). Differentiation is achieved by using different materials, varying task difficulty, providing different levels of instructional support, grouping methods, giving choices, and varying assessment methods (Friend and Bursuck, 2012). Differentiated instruction is popular in inclusive education because it allows working with groups with different abilities and, by nature, meets the requirements for equal access to education and equal instructional opportunities (Savu-Cristescu, 2013; Smit and Humpert, 2012). Most studies have found a small to moderate positive effect of differentiated instruction on students’ achievement (Smale-Jacobse et al., 2019).

Another group of teaching practices that are considered desirable in inclusive classes are various metacognitive strategies. Metacognition is considered a behavioral expression of executive functions, which include planning, organization, working memory, task initiation, set-shifting, impulse control, and self-monitoring (Basham et al., 2020). Metacognitive strategies use knowledge about these cognitive processes and represent an attempt to regulate one’s own learning through planning, monitoring, and evaluation (Erdoğan and Şengül, 2017; Muhid et al., 2020). They also refer to the knowledge of when, where, why, and how to use specific tactics and strategies in the appropriate context (Hattie et al., 1996), i.e., they teach students to understand their way of thinking (Hornby, 2014). Metacognitive skills are also an important part of the body of knowledge, defining a promising approach in inclusive education – The Universal Design for Learning (García-Campos et al., 2020; Sewell et al., 2022), especially in the development of expert learners who want to learn deeply, know how to learn, and how they learn best (Navaitienė and Stasiūnaitienė, 2021).

Therefore, we hypothesize:

*Hypothesis 5:* Student achievement in general knowledge and mathematics will be positively related to how frequently teachers use differentiated instruction and metacognitive strategies.

## 3 Theoretical framework

By observing inclusive education as a continuous process of education system development in such a way that it can accommodate every student and provide them with quality education, and which requires the collaboration of numerous stakeholders at different levels, we believe that Bronfenbrenner’s ecological model is a suitable framework for monitoring students’ academic development in an inclusive environment. Bearing in mind the social nature of inclusive education, researching it, whether as a whole or its individual aspects, should involve determining the relationship between different people and social systems that create and shape inclusive education (Anderson et al., 2014). In considering the ecological structure of educational settings, Bronfenbrenner (1976, 2001) views them topologically as a nested arrangement of structures, each contained in the next one (microsystem, mesosystem, exosystem, macrosystem), which in researching inclusive education allows considering various factors and focusing on the relationship between individual and contextual characteristics (Kamenopoulou, 2016). Adopting this theoretical perspective enables the introduction of a certain order into the wide range of contextual factors that operate at different levels, where identifying factors that operate within or between systems and their roles (facilitating or limiting) allows for a better understanding of inclusive education (Singal, 2006). Thus, various authors worldwide have used Bronfenbrenner’s theoretical concept as a framework for identifying potential barriers and facilitators of inclusion at preschool (see Odom et al., 1996, 2004), as well as school age (see Akbayrak and Douglas, 2022; Okyere et al., 2019; Olechowska, 2020; Panopoulos and Drossinou-Korea, 2020; Pavlović Babić et al., 2018; Ruppar et al., 2017; Tahir et al., 2019; Trang Thu et al., 2022).

With regard to selecting the instruments and study design, Bronfenbrenner’s ecological model, on which the ecology of inclusive education is based, allows the researchers to choose either a quantitative or qualitative approach to the research problem. Furthermore, researchers can use cross-sectional smaller-scope studies, which provide insight into the examined issue at a single point in time, or, on the other hand, employ more extensive longitudinal studies, covering various types and numbers of school environments (Anderson et al., 2014). It is neither realistic nor necessary for a single study to include all possible factors of all systems and their interactions (Bronfenbrenner, 1979). Thus, within the mentioned theoretical approach, researchers should be selective regarding the number of systems and factors within them that will be included in the study (Kamenopoulou, 2016). This paper primarily focuses on the microsystem (classroom level) and the mesosystem (school level).

## 4 Method

### 4.1 The sample

The sample included 331 students from 18 classes attending the third to fifth grade of regular elementary schools. The respondents were students of eight schools located in the urban areas of the Republic of Srpska entity in Bosnia and Herzegovina.

The sample was divided into two subsamples. The first subsample (49.2%) consisted of students attending classes with one student with mild to moderate intellectual disability (CID). The type and degree of intellectual disability were determined by the Expert Committee, which locally assesses the possibilities and gives recommendations for continuing education of children with developmental disabilities. Students with intellectual disability spend all their time in regular classes. The second subsample (50.8%) consisted of students attending classes that did not include students with any type of disability (CNID).

Most respondents were from the third grade (40.5%), followed by those from the fifth grade (38.1%), while the fewest respondents attended the fourth grade (21.5%). With regard to gender, the sample consisted of 48.6% boys and 51.4% girls. Table 1 shows data related to the respondents’ grade and gender in relation to the subsample they belong to.

**Table 1:** Structure of the respondents in relation to gender and age.

Students	CID		CNID		Full sample	
	*n*	*%*	*n*	*%*	*n*	*%*
Gender						
Male	78	23.5	83	25.1	161	48.6
Female	85	25.7	85	25.7	170	51.4
Grade						
Third	67	20.2	67	20.2	134	40.4
Fourth	36	10.9	35	10.7	71	21.6
Fifth	60	18.1	66	19.9	126	38.0

CID, classes with students with ID; CNID, classes without students with ID.

Data on teachers’ attitudes toward inclusion, teachers’ self-efficacy for working in an inclusive environment, and their teaching strategies were obtained from the teachers who taught in the respondents’ classes (nine teachers in each CID and CNID class). All teachers were women, 32–62 years of age (*M* = 41.50, SD = 8.21), with work experience from seven to 31 years (*M* = 15.67, SD = 6.59). The number of students in their classes ranged from 16 to 27 (*M* = 22.50, SD = 3.62).

In addition, while assessing school inclusiveness, all elementary school teachers from the examined schools were included in the sample (*N* = 94). These teachers were 25–64 years of age (*M* = 43.91, SD = 9.65), with 1–39 years of work experience (*M* = 17.99, SD = 9.78). Most of them stated that there were no students with disabilities in their classes (81.9%).

### 4.2 Instruments

We used the *Peabody Individual Achievement Test (PIAT-R)* for the assessment of general knowledge and mathematics, while the variables related to schools and teachers were assessed using the following instruments: *My Thinking About Inclusion Scale (MTAI)*, *Bender Classroom Structure Questionnaire (BCSQ), Teacher Efficacy for Inclusive Practices (TEIP),* and *Inclusive Process Evaluation Scale.*

The MTAI scale is frequently used in examining teachers’ attitudes toward inclusive education (Avramidis and Kalyva, 2007; Galović et al., 2014; Kielblock and Woodcock, 2023; Mucherah et al., 2023). Teacher self-efficacy for working in an inclusive environment is usually assessed using the TEIP scale (Malinen, 2013; McGarrigle et al., 2023; Mohamed Emam and Al-Mahdy, 2020; Savolainen et al., 2012), including the studies in the linguistic area where the research was conducted (Jakubovic and Memisevic, 2024; Matić et al., 2023), while the BCSQ questionnaire is often used to evaluate teaching strategies (Bender and Ukeje, 1989; Bender et al., 2008; Shippen et al., 2011). In addition, the PIAT-R test is among the most commonly used tests for assessing students’ academic skills primarily due to its accuracy when assessing younger participants (kindergarten and primary grades) (Harmer and Williams, 1978). Its application is also found in other studies (Keenan and Meenan, 2014; Slavkovic and Memisevic, 2019; Slavković et al., 2022; Treiman et al., 2024). To assess the level of school incisiveness, we used the *Inclusive Process Evaluation Scale*, which was developed from the well-known instrument in inclusive education called the *Index for Inclusion* (Booth and Ainscow, 2002). The choice of instruments used in this paper was determined by more frequent use by other authors and good metric characteristics.

All selected instruments had satisfactory internal consistency in this study. The only exceptions were two subscales within the *My Thinking About Inclusion Scale* (*Core Perspectives Scale and Classroom Practices*), where reliability was unsatisfactory (*α* = 0.51 in both). Therefore, only the total score on the scale was considered in the presentation of the results. Table 2 presents a detailed description of each used instrument.

**Table 2:** Description of instruments used in the study.

Instruments	Authors	Aim of instrument	Number of subscale and items	Sample responding and scoring	Cronbach’s alpha in our sample
PIAT-R	Markwardt (1989)	Assess the respondents’ general knowledge and achievements in mathematics. This test is used to examine the achievements of respondents 5–22 years of age.	*General information* and *Mathematics*; General information test includes 100 open-ended questions. The Mathematics subtest includes multiple-choice questions.	Each correct answer is valued with one point. Raw scores were used for students’ achievements.	*General information*: α = 0.95 *Mathematics*: *α* = 0.92
MTAI	Stoiber et al. (1998)	Assess teachers’ attitudes toward inclusion	28 items	Respondents give answers on a five-point Likert scale (from 1 – I completely agree to 5 – I completely disagree). The total score on the Scale ranges from 28 to 140, with lower scores indicating more positive attitudes.	*Overall scale*: *α* = 0.84
BCSQ	Bender (1992)	The instrument is used to determine the instructional strategies that teachers use in their work. The questions refer to teaching strategies used in regular classes.	*Individualized instruction* (13 items); *Metacognitive instruction* (11 items)	The subscales consist of Likert-type items with answers ranging from 1 to 5 (from only rarely to almost always). Higher scores indicate more frequent application of teaching strategies.	*Individualized instruction*: α = 0.74 *Metacognitive instruction*: α = 0.72
TEIP	Sharma et al. (2012)	Assess teachers’ self-efficacy for working in an inclusive environment.	Three subscales of six items: *Efficacy in using inclusive instructions*; *Efficacy in collaboration*; *Efficacy in managing behavior*	Respondents answer on a six-point scale (from 1 – I completely disagree to 6 – I completely agree), with a higher score indicating higher self-efficacy.	*Overall scale*: α = 0.90 *Efficacy in using inclusive instructions subscale*: α = 0.81 *Efficacy in collaboration*: α =0.75 *Efficacy in managing behavior*: α = 0.71
Inclusive Process Evaluation Scale	Cottini et al. (2016)	The scale is a tool for assessed school inclusiveness, evaluating and considering various indicators of the inclusive process.	40 items divided into two subscales: *Inclusive organization* and *Inclusive teaching*.	The answers range from 1 (I disagree) to 4 (I agree), and a higher score indicates greater school inclusiveness.	*Inclusive teaching subscale*: α = 0.76 *Inclusive organization:* α =. 90

### 4.3 Research procedure

The research was carried out in several phases. In the first phase, we obtained consent from the Ministry of Education and Culture of the Republic of Srpska to conduct the research in elementary schools. During the second phase, we contacted school representatives, after which data was collected on the presence of children with intellectual disability in regular classes. Also, a document with a detailed explanation of the aim and purpose of the research was delivered to school administrations, along with approval from the Ministry. The third phase included selecting classes with students with intellectual disability and classes with no students with disability. The condition was that the classes were of the same grade and in the same school. In the fourth phase, we contacted teachers who teach the selected classes and agreed on the method of realization of the field part of the research. The teachers introduced the research to the children and their parents, who signed the informed consent. The final phase included data collection. The students were given instructions and detailed explanations about how to complete each instrument. Also, it was pointed out that the research was anonymous.

It should be noted that the described procedure is not without limitations. For example, environmental conditions, such as the fact that the research was conducted in a group, may have influenced the results, as this method can be limited by noise and lack of concentration and involvement in the question content. Although it is more economical, a different approach might have provided more reliable results. Furthermore, data collection through a questionnaire can be a limiting factor because, among other things, it increases the likelihood of socially desirable responses. Also, in Likert-type scales, participants may be inclined to choose extreme responses (selecting the lowest or the highest possible response), which again leads to them not expressing their true attitudes and opinions.

### 4.4 Statistical method

First, the indicators of the skewness and normality of the results’ distribution were examined. The Kolmgorov-Smirnov test showed a normal distribution of the data obtained using the following scales: *Efficacy in using inclusive instructions* (*p* = 0.200) and *Efficacy in collaboration* (*p* = 0.111), *My Thinking About Inclusion Scale* – *MTAI* (*p* = 0.200), as well as the *Individualized instruction* (*p* = 0.189) and *Metacognitive instruction* (*p* = 0.200) subscales. Medians, quartiles, and minimum and maximum values were used to describe significant parameters. The Mann–Whitney U test was used in further data processing.

## 5 Results

### 5.1 Differences in the achievements of students on the general knowledge and mathematics test

No statistically significant difference was found between CID and CNID respondents on the General information test (*p* = 0.242) or the Mathematics test (*p* = 0.190). Table 3 shows descriptive data related to the achievement of both groups on these tests. These results confirm the first research hypothesis.

**Table 3:** Significance of differences between the tested groups of students in relation to their achievements on the general information and mathematics tests.

Scale	Class	*Med.*	*IQR*	*Min.*	*Max.*	*Q1*	*Q3*	U	Z	*p*
General information	CID	31.00	11.00	6.00	58.00	24.00	35.00	12673.500	−1.171	0.242
	CNID	32.50	17.00	9.00	63.00	24.00	41.00			
Mathematics	CID	41.00	19.00	14.00	67.00	34.00	53.00	12552.000	−1.310	0.190
	CNID	45.00	21.00	15.00	76.00	34.00	55.00			

CID, classes with students with ID; CNID, classes without students with ID.

### 5.2 Descriptive parameters of variables related to school and teachers

Table 4 shows descriptive indicators of school inclusiveness. Most teachers believe that the schools where they work have a high level of inclusiveness with regard to teaching (Q1 = 64.49, Q3 = 72.46), while they assess school organization as somewhat lower but still within medium and high values (Q1 = 55.09, Q3 = 69.38).

**Table 4:** Descriptive parameters of school inclusiveness.

Scale	*Mdn*	*IQR*	*Min.*	*Max.*	*Q1*	*Q3*
School inclusiveness – organization	62.98	14.28	52.29	73.30	55.09	69.38
School inclusiveness – teaching	66.64	7.97	63.89	74.33	64.49	72.46

Descriptive parameters of independent variables, i.e., the variables that refer to teachers in CID, are shown in Table 5. Most teachers teaching CID classes feel prepared to work in an inclusive environment. They assess their self-efficacy as high in relation to behavior management (Q1 = 28.50, Q3 = 32.50) and cooperation with other participants in the inclusive process (Q1 = 28.00, Q3 = 32.00), while they are somewhat less confident in their ability to teach in this environment (Q1 = 23.00, Q3 = 26.50). Also, teachers relatively frequently use individualized (Min = 44.00; Max = 63.00) and metacognitive teaching strategies (Min = 42.00, Max = 49.00), with metacognitive strategies still being a bit more frequent. CID teachers’ attitudes toward inclusive education range from positive to slightly negative (Min. = 56.00, Max. = 105.00), most of them being slightly positive or neutral (Q1 = 63.00, Q3 = 91.00).

**Table 5:** Descriptive parameters of independent variables in CID.

Scales	*Mdn*	*IQR*	*Min.*	*Max.*	*Q1*	*Q3*
Self-efficacy – instruction	25.00	3.50	22.00	30.00	23.00	26.50
Self-efficacy – collaboration	31.00	4.00	27.00	36.00	28.00	32.00
Self-efficacy – behavior	29.00	4.00	27.00	34.00	28.50	32.50
Metacognitive strategies	44.00	4.50	42.00	49.00	42.50	47.50
Individualized strategies	49.00	6.50	44.00	63.00	44.50	51.00
Attitudes – inclusive education	80.00	28.00	56.00	105.00	63.00	91.00

Table 6 shows the results of assessing independent variables in CNID. Teachers of these classes also highly rate their competencies for working in an inclusive environment, although to a lesser extent. Just like CID teachers, they also express higher self-efficacy regarding collaboration with other participants of the inclusive educational process (Q1 = 27.50, Q3 = 29.50) and behavior management (Q1 = 28.50, Q3 = 31.50) than regarding teaching in an inclusive class (Q1 = 22.00, Q3 = 25.50).

**Table 6:** Descriptive parameters of independent variables in CNID.

Scales	*Mdn*	*IQR*	*Min.*	*Max.*	*Q1*	*Q3*
Self-efficacy–instruction	24.00	3.50	19.00	27.00	22.00	25.50
Self-efficacy – collaboration	29.00	2.00	21.00	31.00	27.50	29.50
Self-efficacy–behavior	29.00	3.00	27.00	35.00	28.50	31.50
Metacognitive strategies	44.00	6.00	40.00	48.00	41.00	47.00
Individualized strategies	45.00	8.00	42.00	52.00	42.00	50.00
Attitudes–inclusive education	79.00	15.50	56.00	85.00	68.50	84.00

CNID teachers also more frequently use metacognitive (Q1 = 41.00, Q3 = 47.00) than individualized teaching strategies (Q1 = 42.00, Q3 = 50.00). Although the range of responses regarding their attitudes toward inclusive education is narrower than in CID teachers and ranges from clearly positive to neutral (Min. = 56.00, Max. = 85.00), i.e., there are no negative attitudes, most attitudes in this subsample are neutral or slightly positive (Q1 = 68.50, Q3 = 84.00).

### 5.3 The relationship between student achievement and school and teacher-related variables

Raw scores were used to correlate student-related variables with teacher-related variables (*TEIP, MTAI, Individualized instruction,* and *Metacognitive instruction*) and school-related variables (*Inclusive Process Evaluation Scale*). Teachers in both subsamples and schools were divided into two groups based on achievements on the mentioned instruments. One group includes the results above the median, representing better achievements. The other group includes the results below the median (lower achievements group). The only exception is the Scale for assessing attitudes toward inclusive education, where a higher score indicates more negative attitudes.

In CID, students attending more inclusive schools in terms of organization had significantly better achievements on the *General knowledge test* (*p* = 0.000, *d* = 0.28). Furthermore, students attending classes taught by teachers with higher self-efficacy for teaching (*p* = 0.000, *d* = 0.28) and collaboration (*p* = 0.024, *d* = 0.18) in an inclusive environment, as well as those whose teachers more often used both metacognitive (*p* = 0.002, *d* = 0.24) and individualized (*p* = 0.017, *d* = 0.19) teaching strategies, were more successful when testing general knowledge. According to Cohen’s criterion, the effect size is small to medium (Pallant, 2017).

With regard to CID students’ achievement on the *Mathematics* test, students attending more inclusive schools in terms of teaching (*p* = 0.000, *d* = 0.28) and work organization (*p* = 0.000, *d* = 0.40) had higher achievements, as well as students whose teachers had higher self-efficacy for teaching (*p* = 0.000, *d* = 0.36) and collaboration (*p* = 0.001, *d* = 0.26) and used metacognitive teaching strategies more often (*p* = 0.000, *d* = 0.33). The obtained values of Cohen’s d indicate that these variables have a medium effect on mathematics test achievements. That is, the results indicate that attending a school with higher inclusiveness in terms of organization, as well as higher self-efficacy for teaching and more frequent use of metacognitive teaching strategies, play a somewhat greater role in academic achievements (Table 7).

**Table 7:** Significance of differences in student achievement on general knowledge and mathematics tests in relation to the examined factors in CID.

Scales	General knowledge			Mathematics		
	U	Z	*p*	U	Z	*p*
School inclusiveness – organization	2235.000	−3.566	**0.000**	1762.000	−5.139	**0.000**
School inclusiveness – teaching	2512.000	−1.773	0.076	1986.000	−3.604	**0.000**
Self-efficacy – teaching	1508.500	−3.541	**0.000**	1245.000	−4.566	**0.000**
Self-efficacy – collaboration	2616.500	−2.257	**0.024**	2282.500	−3.370	**0.001**
Self-efficacy – behavior	2012.000	−0.922	0.356	2135.000	−0.425	0.671
Metacognitive strategies	1777.500	−3.145	**0.002**	1492.500	−4.210	**0.000**
Individualized strategies	2333.500	−2.394	**0.017**	2503.500	−1.802	0.072
Attitudes – inclusive education	2807.500	−0.381	0.703	2426.000	−1.733	0.083

The bolded values in the table indicate statistically significant results.

CNID students attending schools with a higher degree of inclusive organization also had better achievements on general knowledge (*p* = 0.047, *d* = 0.15) and mathematics (*p* = 0.000, *d* = 0.28) tests, with small and medium effect sizes. These results suggest that although the differences in achievements are statistically significant, the effect size varies, being somewhat higher regarding achievements in mathematics. Furthermore, CNID students whose teachers had higher self-efficacy for behavior management in inclusive classes were significantly more successful on both tests (Mathematics: *p* = 0.000, *d* = 0.30; General knowledge: *p* = 0.030, *d* = 0.17). This indicates that teacher self-efficacy can have a more significant role in student achievement on the mathematics test compared to general knowledge.

However, in CNID, teachers’ self-efficacy for teaching in an inclusive environment was related to negative academic outcomes in some aspects. Thus, students whose teachers considered their own self-efficacy for teaching in inclusive classes higher had significantly lower achievements in mathematics (*p* = 0.038, *d* = 0.16), while students whose teachers believed to have higher competencies for collaboration in an inclusive environment had significantly lower achievements in the general knowledge test (*p* = 0.034, *d* = 0.16). Finally, CNID students whose teachers had worse attitudes toward inclusive education had better achievements on both tests (General knowledge: *p* = 0.002, *d* = 0.23; Mathematics: *p* = 0.000, *d* = 0.28). In all described relationships, the effect sizes were within small and medium values. Also, teacher attitudes toward inclusive education play a slightly greater role. Thus, Hypotheses 2, 3, 4, and 5 were partially supported (Table 8).

**Table 8:** Significance of differences in student achievement on general knowledge and mathematics tests in relation to the examined factors in CNID.

Scales	General knowledge			Mathematics		
	U	Z	*p*	U	Z	*p*
School inclusiveness – organization	2897.000	−1.990	**0.047**	2362.500	−3.688	**0.000**
School inclusiveness – teaching	3167.000	−0.753	0.451	2869.000	−1.717	0.086
Self-efficacy – teaching	2788.000	−1.703	0.089	2673.000	−2.080	**0.038**
Self-efficacy – collaboration	2847.500	−2.124	**0.034**	3042.000	−1.506	0.132
Self-efficacy – behavior	1899.000	−2.166	**0.030**	1457.000	−3.843	**0.000**
Metacognitive strategies	3191.500	−0.567	0.571	3074.000	−0.949	0.343
Individualized strategies	3182.000	−1.060	0.289	3479.500	−0.114	0.909
Attitudes – inclusive education	2210.000	−3.035	**0.002**	2032.500	−3.636	**0.000**

The bolded values in the table indicate statistically significant results.

## 6 Discussion

The most important finding of this research is the absence of significant differences between the achievements of typically developing students attending CID and CNID in both general knowledge and mathematics. In general, typically developing students in CNID achieved somewhat better results in both tests, but the difference was not statistically significant.

The obtained results are in accordance with most studies that found neutral effects of inclusive education on the academic outcomes of typically developing students (e.g., Farrell et al., 2007; Kalambouka et al., 2007; Ruijs, 2017). In other words, despite the sometimes-expressed concern (e.g., Ahsan et al., 2012; Almotairi, 2013; Dignath et al., 2022; Tafa and Manolitsis, 2003), inclusive education has no negative effects on the academic achievement of typically developing students.

The inclusive organization of the schools is related to differences in students’ general knowledge and mathematics achievements in both CID and CNID classes. Students who attended more inclusive schools in terms of organization had better achievements. The obtained results are in accordance with the attitudes of authors who believe that inclusive schools are the most effective means of achieving educational goals for all students (Hoppey and McLeskey, 2014) and that these schools provide quality education to all students according to their needs (Messiou, 2017; Opertti et al., 2014; Óskarsdóttir et al., 2020). On the other hand, school inclusiveness in terms of teaching is only related to higher general knowledge achievements of CID students. The relation to academic outcomes was also expected in CNID classes and mathematics. The absence of these relations might be associated with the fact that most tested teachers believed that their schools had a high level of inclusive teaching, and, thus, there was not enough variability in this regard.

In CID, teachers’ self-efficacy for collaboration and teaching in an inclusive environment was positively related to the acquisition of general knowledge and mathematics, while confidence in their competencies regarding behavior management was not significantly related to the examined academic domains. In their metanalytical study, Kim and Seo (2018) explain similar findings by the context-specific nature of teachers’ self-efficacy. Tschannen-Moran et al. (1998) believe that teacher efficacy in terms of teaching strategies and student engagement depends on the teacher’s confidence in their own teaching methods and ability to motivate students, which are factors closely related to academic achievement. On the other hand, according to these authors, teacher efficacy in behavior management can be less relevant to students’ academic achievement.

In CNID, the relationship between self-efficacy for working in an inclusive environment and knowledge acquisition is more complex. In contrast to CID, the self-perceived teachers’ ability to manage classroom behavior was the only one that significantly positively related to students’ achievements in general knowledge and mathematics. Furthermore, self-efficacy for collaboration (general knowledge) and self-efficacy for teaching (mathematics) were negatively related to student knowledge.

It is possible that teachers in CID assessed their self-efficacy for inclusive education more realistically, based on the experience in the class attended by a student with disability, and that they successfully implemented it through collaboration and more inclusive teaching, which was reflected in higher student achievement. In contrast, despite similar confidence in their self-efficacy for working in an inclusive environment, teachers in CNID possibly teach in a more traditional manner (e.g., more frontal work, less differentiation), which, according to the obtained results, was, for example, reflected in the somewhat scarcer implementation of individualized instruction. With regard to small effect sizes, we should leave the possibility that the negative relation of self-efficacy for teaching and collaboration with student achievement is just an artifact.

Even though teachers in both educational environments often used both individualized and metacognitive strategies, although the metacognitive ones were a bit more frequent, their effects on student academic achievement were not equal. In CID, students whose teachers used both strategies more often had higher achievements on general knowledge tests, while only the frequency of metacognitive strategies was related to better achievements in mathematics. In addition, the relationship with metacognitive strategies had higher effect sizes. These results suggest that the practices aimed at adapting the curriculum and instruction to student differences (e.g., differentiation and individualization) are important for success in inclusive classes. However, it is equally, if not more, important that teachers instruct students on how to gain control over the learning process and gradually become more successful in mastering their own mental processes (Peklaj, 2015; Zhu, 2015).

Contrary to the results obtained in CID, the frequency of individualized and metacognitive strategies was not related to the differences in student knowledge in CNID. The application of these strategies may be especially important for success in classes with a greater diversity of students with regard to developmental characteristics (Deunk et al., 2018; Hornby, 2014; Jung et al., 2018; Smale-Jacobse et al., 2019; Vázquez-Chaves, 2015). On the other hand, the frequency of using these strategies is also significant in more homogenous classes (Bernard et al., 2019; Muhid et al., 2020; Perry et al., 2019). However, at least in our study, not so much as to make a difference in student academic achievement. Vettori et al. (2018) also found a non-significant relation between metacognitive skills/strategies and academic achievements of Italian middle-school students. They believe that such results indicate the controversial nature of the relationship between these two variables.

Attitudes of both groups of teachers (CID and CNID) toward inclusive education mainly ranged from neutral to slightly positive, although the attitudes of CNID teachers were more homogenous compared to their CID colleagues. The results obtained are in the middle of the range of results from recent review studies. Lindner et al. (2023) found that primary school teachers had neutral or ambivalent attitudes, while Spanish teachers had generally positive attitudes, although some of them also had ambivalent attitudes (Lacruz-Pérez et al., 2021). At the ends of the spectrum are reviews that identify clearly positive (Guillemot et al., 2022; Van Steen and Wilson, 2020) or negative (de Boer et al., 2011; Van Mieghem et al., 2020) teacher attitudes toward inclusive education. However, regarding the relation of these attitudes with student academic achievement, the obtained results are quite unexpected. Not only were the academic achievements of students in CID unrelated to their teachers’ attitudes toward inclusive education, but teachers’ attitudes in CNID were negatively related to their students’ achievements on general knowledge and mathematics tests. Although the effect sizes in both relationships are small, the obtained results are contrary to what was expected (Asres, 2019; Gal et al., 2010). However, even though research on the direct relationship between attitudes toward inclusive education and student academic achievement is scarce, as noticed by other authors (Heyder et al., 2020), there are studies whose results are similar to those obtained in our study (see Hornstra et al., 2010; Kamran et al., 2023).

The obtained range of attitudes (which are not clearly positive) may express teachers’ reservations about inclusive education, which is then reflected in the application of inclusive teaching practices that affect student achievement (Hellmich et al., 2019; Jordan et al., 1997; Pit-ten Cate et al., 2018; Schwab and Alnahdi, 2020; Sharma and Sokal, 2016). This could explain the absence of a relationship between teachers’ attitudes toward inclusive education and students’ academic achievements in CID, as well as the negative relationship in CNID, despite the fact that teachers report frequent use of metacognitive strategies and individualized instruction.

## 7 Theoretical implications

Our research determined that teachers reported higher self-efficacy when teaching more successful students, especially those who taught math and science. Also, teachers did not feel equally efficient in all situations and learning tasks, which supports the integrative self-efficacy model proposed by Tschannen-Moran et al. (1998), according to which teacher self-efficacy varies depending on the teaching task and context. These two dimensions are also related to the assessment of available resources that facilitate learning, as well as to the self-perception of teaching competence.

Although self-efficacy and attitudes are two highly related constructs that influence teacher behavior and that, according to some authors, should be examined together (Sharma and George, 2016), the obtained relationships between attitudes and academic outcomes (or the absence of this relationship) raise some questions. For example, are teacher attitudes toward inclusive education important for student achievement in an inclusive environment, despite the existing consensus (e.g., Avramidis and Norwich, 2002; Mouchritsa et al., 2022; Saloviita, 2020a)? As a widely used method, are questionnaires appropriate for examining attitudes because they favor the human tendency to present themselves as socially or politically correct, which can interfere with expressing negative attitudes and prejudices (Akrami et al., 2006)? Thus, one study reports that the perceived attitude of the organization conducting the research explained over 20% of the variance (more variance than all other examined predictors together) of the general population’s attitudes toward inclusive education (Lüke and Grosche, 2018). However, the obtained results primarily indicate the complexity of the factors affecting student academic achievements and the complex relationship between these achievements and the attitudes toward inclusive education.

Identifying various factors in this research, which act both within and between different systems in the ecological structure of the educational environment, allowed for a better understanding of the connection between an individual (student) and the context in which they develop. Our results shed light on part of the conceptual framework of Bronfenbrenner’s ecology systems model, suggesting that certain factors at the microsystem (teacher self-efficacy and the use of metacognitive and individualized teaching strategies) and the mesosystem (school inclusiveness in terms of organization and teaching) levels positively affect specific academic outcomes of students. These results expand the existing body of knowledge on potential barriers and factors that facilitate inclusion at various levels of ecological systems. Pavlović Babić et al. (2018) identify factors at the micro and mesosystem levels (classroom and school, respectively) that contribute to the successful implementation of inclusive education in Serbia. Direct interaction between students and teachers, primarily expressed through the teachers’ willingness and competence to adapt teaching methods to the needs of children, was the most influential factor at the microsystem level, while open communication among school staff was an important factor at the mesosystem level when establishing a positive classroom climate. Tahir et al. (2019) found similar results, identifying the physical organization of the learning space and resources, as well as classroom teaching practices, as important factors at the microsystem level, and the significance of collaboration among school staff, as well as with parents, as an important factor in promoting learning at the mesosystem level. However, unlike previously mentioned studies, as well as most other studies where the theoretical framework was applied in examining inclusive education for students with special educational needs (see Akbayrak and Douglas, 2022; Okyere et al., 2019; Olechowska, 2020; Panopoulos and Drossinou-Korea, 2020; Ruppar et al., 2017; Trang Thu et al., 2022), our research extends this framework to the inclusion of all students.

## 8 Managerial implications

School principals and administration aiming for school development toward greater inclusiveness could benefit from the results of this study. School management needs to be aware of the classroom and school characteristics that contribute to positive academic outcomes for all students and direct the management practices so that they positively affect these characteristics. Among other things, this would involve engaging and coordinating teachers, students, and parents toward achieving mutual educational goals (Óskarsdóttir et al., 2020), creating an educational system in which teachers are supported and ready to explore effective teaching methods that would benefit all students (Ainscow and Sandill, 2010), as well as ensuring professional development for teachers who play a key role in the academic achievement of all students (Szumski et al., 2017). We believe that the data obtained in this study can also be useful for decision-makers at the local or national level regarding the need for school organization toward greater inclusiveness, as well as the need for developing teacher training programs. Teacher education university programs, as well as continuous professional development programs for teachers, should include those teaching methods that contribute to better academic achievements of all children, which is particularly important in Bosnia and Herzegovina, where insufficient pre-service and in-service teacher training is stated as one of the barriers to inclusive education (Čelebičić and Jovanović, 2021).

## 9 Limitations and future research avenues

Although the obtained results, in general, do not register the negative effects of attending classes with peers with intellectual disability on the academic achievements of typically developing students, we should be careful when interpreting them due to the existing limitations. One of the limitations refers to the convenience sample of the respondents and schools included in the research, which requires verifying the results on a larger sample of both students and teachers. In addition, we did not consider the possibility of a differentiated effect of inclusive education on students with different levels of achievement. Results of some studies have found that students with lower achievements can benefit more from inclusive teaching (Huber et al., 2001). This scarcely researched question is important since the obtained neutral effects can result from a differentiated effect (e.g., low-achieving students benefit from inclusion, high-achieving students are disadvantaged, and the overall result is neutral).

Our study focused only on certain factors within the micro and meso systems that are not independent of what occurs in other systems, such as the exo and macro systems, or even within other aspects of the micro and meso systems (see Tahir et al., 2019), which could be addressed in future research.

With regard to that, expanding the research with qualitative data, i.e., applying a mixed-method approach, would allow for a more refined analysis of certain variables and a better understanding of the interactions within and between the mentioned ecosystems.

The cross-sectional nature of the study does not enable us to track changes over time, which would be significant given that positive or neutral effects of inclusive education on academic achievement are usually observed in lower grades, while neutral or negative effects are more common in higher grades (Kart and Kart, 2021). Thus, a longitudinal study would enable the identification of not only changes and long-term effects but also causal relationships or reciprocity in the relationships between different aspects of the observed ecological systems, as well as potential mediators and moderators within those relationships.

## 10 Conclusion

This research did not find significant differences in the achievements of typically developing students when assessing their general knowledge and mathematics with regard to whether they attended CID or CNID, which confirms the results of most previous studies indicating that the presence of students with disabilities does not lower the achievements of typically developing students (e.g., Dessemontet and Bless, 2013; Farrell et al., 2007; Kalambouka et al., 2007; Ruijs, 2017). In both groups, students with better academic achievements attended more inclusive schools in terms of organization and teaching. On the other hand, the results obtained by examining the relationship between academic outcomes and teacher variables are often confusing. Thus, in CID, we determined better achievements in students whose teachers were characterized by higher self-efficacy with regard to teaching and collaboration, as well as more frequent application of metacognitive and/or individualized teaching strategies. In contrast, in CNID, lower academic achievements were determined in students whose teachers expressed higher self-efficacy for teaching and more positive attitudes toward inclusive education. Only higher self-efficacy regarding behavior management was related to positive academic outcomes. All this implies that the relationship between teacher characteristics and student academic achievements in an inclusive school is a complex phenomenon that requires further research. Given the results obtained, educational practice should be directed toward creating a system that would provide teachers with support through collaboration and professional development in teaching strategies that contribute to positive academic outcomes for all students.

## Data Availability

The raw data supporting the conclusions of this article will be made available by the authors, without undue reservation.
